# P-793. Long-term Outcomes of *Mycobacterium avim* complex Pulmonary Disease in Kyushu, Japan: A Retrospective Multicenter Study

**DOI:** 10.1093/ofid/ofae631.985

**Published:** 2025-01-29

**Authors:** Shotaro Ide, Takahiro Takazono, Shinpei Morimoto, Masataka Yoshida, Kazuaki Takeda, Naoki Iwanaga, Naoki Hosogaya, Kosaku Komiya, Koichi Izumikawa, Akitsugu Furumoto, Katsunori Yanagihara, Hiroshi Mukae

**Affiliations:** Nagasaki University Hospital, Nagasaki, Nagasaki, Japan; Nagasaki University Graduate School of Biomedical Sciences, Nagasaki, Nagasaki, Japan; Nagasaki University, Nagasaki, Nagasaki, Japan; Nagasaki University Hospital, Nagasaki, Nagasaki, Japan; Nagasaki University Hospital, Nagasaki, Nagasaki, Japan; Nagasaki University Hospital, Nagasaki, Nagasaki, Japan; Nagasaki University, Nagasaki, Nagasaki, Japan; Oita University Faculty of Medicine, Yufu, Oita, Japan; Nagasaki University, Nagasaki, Nagasaki, Japan; Nagasaki University Hospital, Nagasaki, Nagasaki, Japan; Nagasaki University, Nagasaki, Nagasaki, Japan; Nagasaki University, Nagasaki, Nagasaki, Japan

## Abstract

**Background:**

Recently, the number of patients with nontuberculous mycobacterial pulmonary disease (NTM-PD) has increased worldwide. The most common causative organism in Japan is the *Mycobacterium avium* complex (MAC). The incidence of MAC pulmonary disease (MAC-PD) is higher than that in other countries, and deaths from MAC-PD are increasing. Complex cases involving radiological fibrocavitary patterns and pulmonary aspergillosis have been reported as poor prognostic factors; however, available evidence is limited. To address this gap, we initiated a retrospective, multicenter study in Kyushu, Japan, focusing on the long-term prognosis of patients five years after the diagnosis of MAC-PD. The primary objective of this study was to investigate the factors that influence the outcomes of these patients by employing data accessible at the time of their initial diagnosis.

**Methods:**

This was a retrospective cohort study of patients aged ≥ 18 years with newly diagnosed NTM-PD who visited 18 medical centers in Kyushu, Japan, between 2010 and 2017. Patient data were collected from medical records by respiratory experts and recorded using the EDC.

**Results:**

The study enrolled 1317 patients with NTM-PD, of whom 1214 had MAC-PD. Patients with insufficient information were excluded, and 856 patients were analyzed: 546 (63.8%) with a non-cavitary nodular bronchiectatic (NB) pattern and 194 (22.7%) with a cavitary NB or fibrocavitary pattern. 107 (12.5%) died from all-cause by 5 years after diagnosis and 137 (16.0%) by 10 years; in the group that died after 5 years, the following were detected as risk factors in multivariate analysis: male sex (OR = 2.90, 95% confidential interval 1.84-4.56), age ≥ 70 (OR = 2.20, 95%CI 1.33-3.63), lobes with cavities ≥ 2 lobes (OR = 2.16, 95%CI 1.07-4.36), albumin < 3.5 g/dL (OR = 3.68, 95%CI 2.29-5.91), and oral corticosteroids (OR 1.97, 95%CI 1.04-3.73).

**Conclusion:**

Male sex and fibrocavitary patterns have been reported to be poor prognostic factors for MAC-PD. In our study, we found that cavitary formation in more than two lobes, lower albumin levels, and oral corticosteroids were newly considered poor prognostic factors. We believe that our findings may help determine treatment induction and predict the prognosis of MAC-PD.

**Disclosures:**

**Hiroshi Mukae, M.D., Ph.D.**, AstraZeneca: Lecture fees|Gilead Sciences: Lecture fees|GSK: Lecture fees|MSD: Advisor/Consultant|MSD: Lecture fees|Pfizer: Lecture fees|Shionogi & Co., Ltd.: Advisor/Consultant|Shionogi & Co., Ltd.: Grant/Research Support|Shionogi & Co., Ltd.: Lecture fees

